# Influence of patients’ sex on radiomics-based predictions of lung artery thrombus

**DOI:** 10.1007/s11548-026-03704-1

**Published:** 2026-06-04

**Authors:** Lea Ehrhardt, Georg Hille, Ehsan Amini, Alexey Surov, Jan Borggrefe, Patrique Fiedler, Sylvia Saalfeld

**Affiliations:** 1 Institute of Biomedical Engineering and Informatics, Technische Universität Ilmenau, Ilmenau, Germany; 2 Department of Radiology, Neuroradiology and Nuclear Medicine, Johannes Wesling University Hospital, Ruhr University Bochum, Minden, Germany; 3 Department of Medical Informatics and Statistics, University Hospital Schleswig-Holstein, Campus Kiel, Kiel, Germany

**Keywords:** Radiomics, Pulmonary embolism, Thrombus, Troponin, Mortality, Classification, Sex

## Abstract

**Purpose:**

This study investigates possible differences of female and male patients’ data in regard to the prediction of mortality and troponin level of acute pulmonary embolism (APE) patients. Radiomics features are utilized to gain insights of sex-specific image properties, which should support APE diagnosis and treatment.

**Methods:**

A database of 200 manually segmented APE patients’ computed tomographic data (100 female and 100 male) was used for the prediction of 30-day mortality and biomarker troponin level. The radiomics features were extracted from the segmented thrombi and reduced via correlation analysis and 12 feature selection followed each by 12 classification methods. For comparison, the weighted F1-Score, Matthew’s Correlation Coefficient, and balanced accuracy were used. For assessment of generalizability, the study was extended to 427 automatically selected datasets.

**Results:**

Sex-dependent differences have been found in the resulting metrics and highest achieving feature selection and classification methods. Not stratifying the dataset by sex resulted in lower performance compared to the sex-stratified male and female only calculations. The subgroup of female patients achieved up to 31.04 % higher metric values compared to the other cohorts. Different thrombus properties contributed to the prediction based on the patients’ sex as well.

**Conclusion:**

In this study, we analyzed possible correlations between sex of APE patients and mortality and troponin levels as well as radiomics features. Our analysis identified an influence of sex on the prediction task and image feature selection. Female patient data especially had higher predictive potential, which could help for further sex-stratified analysis of APE.

## Introduction

Acute pulmonary embolism (APE) is a cardiovascular disease, which is widespread and shows high mortality rates [1]. Diagnostic indicators like right ventricle enlargement [2] and inferior vena cava reflux [3], which can be derived from CT pulmonary angiogram images, are normally used to predict medical outcomes like the 30-day mortality (30d-mortality). Multiple studies have successfully demonstrated the potential of radiomics features to support the prediction of the patients mortality [4, 5]. Our previous work confirmed this possibility additionally for the biomarker troponin [6], which is important for the diagnosis and monitoring of APE. The prospect to combine these features with tomographic image data represents a potential for better diagnosis and treatment processes of patients [7]. Symptoms and risk factors of diseases as well as their outcomes and progressions, which are significant for their diagnosis and treatment, are proved to be correlated with different factors, one of these often being the sex of the patient [8]. These differences in representation are not thoroughly known and investigated, due to women being mostly underrepresented in clinical studies even until today. Moreover, their different clinical portrayal in certain cases has only been studied more profoundly in recent years [9]. This limited understanding of these variations can result in difficulties regarding diagnosis and treatment of patients and therefore remains a relevant question in the medical and clinical research [8]. In the context of APE, previous studies regarding differences of female and male patients reported contradictory results. Newman et al. [10] found out that there are no sex-stratified distinctions between the pulmonary embolism (PE) risk category, but also that the female patients’ data can be used to predict in-hospital mortality due to higher risk. Tian et al. [11] also concluded a higher mortality for female patients, but conducted that there were no significant correlations between clinical symptoms and sex in regard to in-hospital mortality. This finding is similar to Zhan et al. [12], who found no clear sex-stratified differences for the mortality of the two sexes. On the other hand, Mesinovic et al. [13] stated that female patients exhibit a slightly lower mortality rate and that sex could be an important feature for its prediction. Teodoru et al. [14] associated the patients’ sex with the mortality rate and specified sex presenting possible influence on PE presentation and outcome. Female patients displaying lower mortality rates was also a finding by Tejero et al. [15]. However, their results showed no distinct possibility for sex to become an independent feature for mortality predictions. These studies do not provide certainty regarding influence of patients’ sex on the prediction task in APE. Therefore, this work investigates the impact of sex on radiomics-based prediction of mortality and troponin levels.

**Fig. 1:**
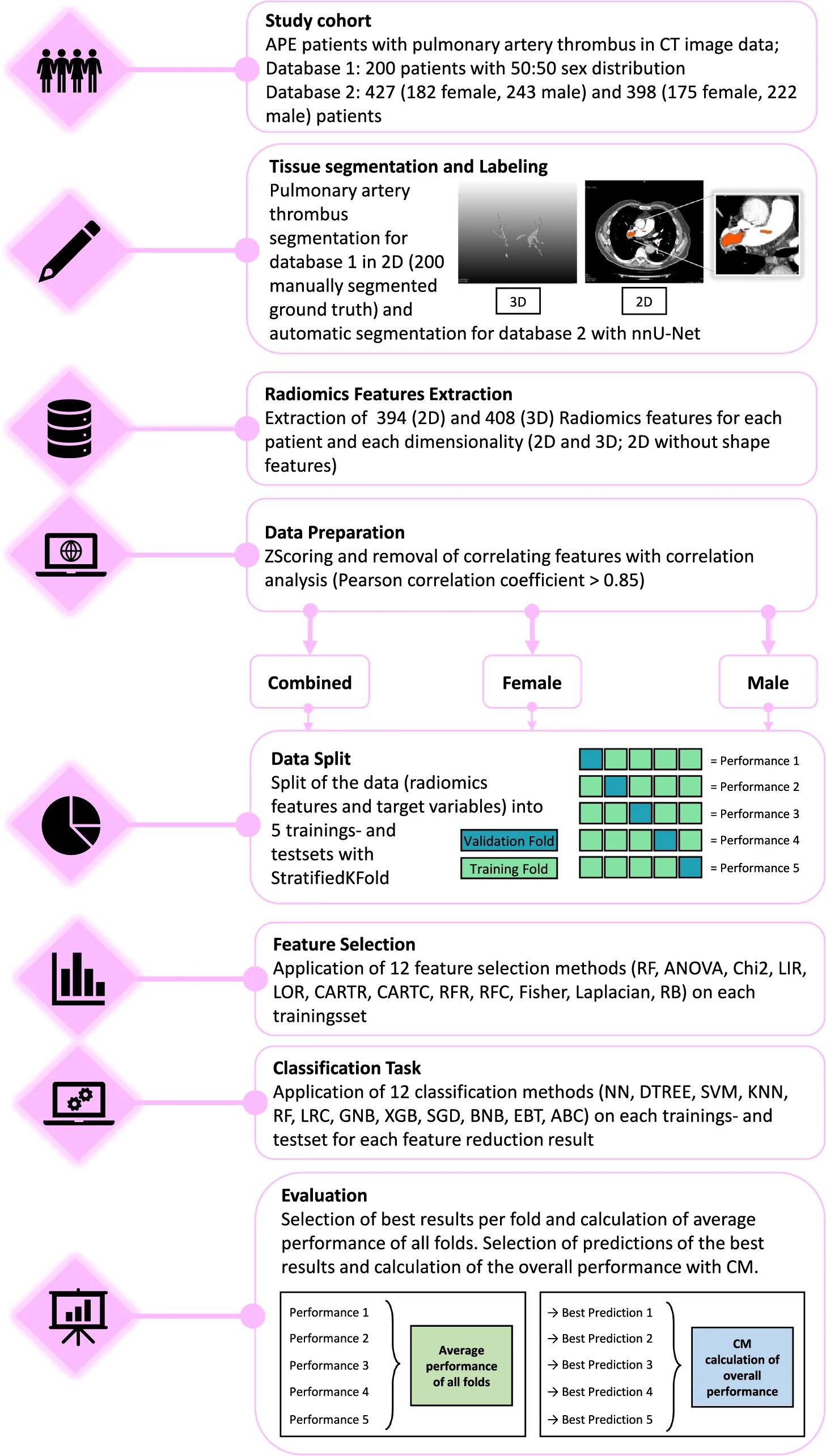
Applied workflow for 200 manually (database 1) and over 400 automatic segmented patients’ data (database 2)

**Table 1:** Highest mean metric values for both target variables, both dimensions, and for the three cohorts of $\textrm{DB}_{1}$

	cohort	cohort	cohort	cohort	cohort	cohort
	${run2D}_{mort}$			${run3D}_{mort}$		
weighted F1	0.9418	0.8941	0.86375	**0.9626**	0.8892	0.8671
MCC	0.4681	0.6167	0.3803	**0.6685**	0.6195	0.3581
balanced accuracy	0.734	0.776	0.5914	**0.8245**	0.8168	0.64095

	${run2D}_{mort}$			${run3D}_{mort}$		
weighted F1	0.8531	0.8513	0.8128	**0.8676**	0.7921	0.7997
MCC	0.6053	0.5958	0.4974	**0.6156**	0.5644	0.4764
balanced accuracy	**0.8093**	0.7857	0.7495	0.7893	0.7813	0.7443

Bold highlights the highest value

**Fig. 2:**
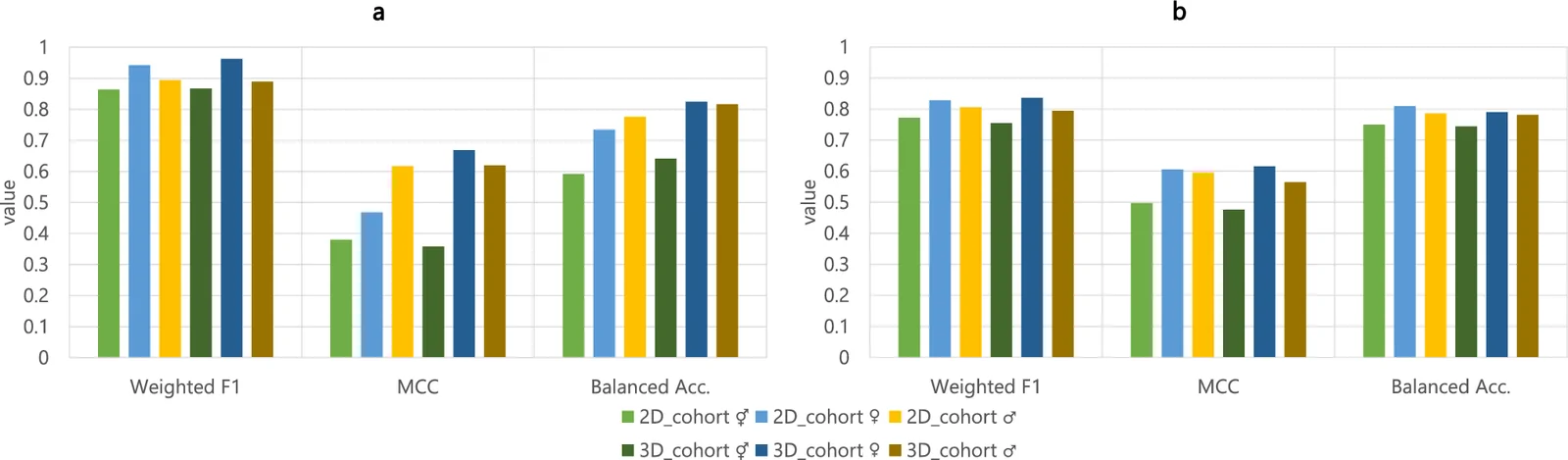
Highest mean values for each metric over three repetition runs. Shown are the results for the three cohorts for **(a)** $run_{mort}$ and **(b)** $run_{trop}$

## Materials and methods

The initial workflow was clarified in our previous work [6]. Its accustomed machine learning workflow and the additional changes will be explained in the following and are illustrated in Fig. 1.

### Medical image dataset and preparation

The processed medical images were part of a retrospective study approved by the institutional review board of the Otto-von-Guericke University of Magdeburg (Nr. 145/21, Ethics Committee, Otto-von-Guericke University of Magdeburg, Magdeburg, Germany). The medical dataset comprised 427 APE patients. More details regarding acquisition and patient information can be found in previous works [4, 6]. From this dataset, we curated a subset of 200 patients’ computed tomography (CT) data randomly, consisting of exactly 100 female and 100 male individuals (database 1, $\textrm{DB}_{1}$). The dataset size was limited by the completeness of patient information and the required manual effort. For inclusion, each patient required information on 30d-mortality and troponin levels. In terms of manual effort, a field expert performed a manual segmentation of the lung thrombus for each case, which was subsequently reviewed by an experienced radiologist. An illustrated example is shown in Fig. 1. A second database curation ($\textrm{DB}_{2}$) was performed to enhance generalizability by maximizing patient inclusion and applying an automated segmentation approach, which is described in [16]. The automatic segmentation via nnU-Net [17] resulted in a Dice similarity coefficient of up to 90% compared to the ground truth of 200 manually segmented thrombi. The medical images used for the training of this network were acquired from the same study and consisted of all patients with available mortality and troponin level information. For the enlarged $\textrm{DB}_{2}$, ground truth segmentation were not available for all patient cases; however, the automatically produced segmentation masks were considered sufficient for radiomics parameter extraction. As a data inclusion criterion, a minimal thrombus size of 0.5 cm$^3$ volume was chosen to guarantee the radiomics features could be extracted correctly from the segmentation masks. As separate target variables, the 30d-mortality and biomarker troponin were selected. Troponin is a biomarker used for instance for APE risk stratification and was additionally chosen to identify possible correlation to the radiomics features based on the patients’ sex. Troponin levels were converted to binary values via the median due to the data being not normally distributed. This resulted in 427 (182 female, 243 male) for the mortality and 398 (175 female, 222 male) patients for the troponin analysis.

### Radiomics workflow

$\textrm{DB}_{1}$ was utilized and used as the main focus in this sex-stratified analysis due to being validated and consisting of an even split of female and male patients for the mortality and troponin prediction each. This database as well as the enlarged $\textrm{DB}_{2}$ was divided into three cohorts each: female only patient data (), male only patient data (), and the not sex-stratified combined patient data (). In total, there were 12 individual runs: four prediction evaluations ($run2D_{mort}$, $run2D_{trop}$, $run3D_{mort}$, $run3D_{trop}$) for each cohort. The same number of iterations was applied to the enlarged $\textrm{DB}_{2}$. Each analysis was implemented separately from each other. Afterward, the radiomics features were calculated with 381 features being extracted in Python via the PyRadiomics function [18]. The classes from which the features were extracted included the same as in our previous work [6]. A correlation analysis (Pearson correlation coefficient > 0.85) and ZScoring were additionally applied on the features for the removal of associating characteristics [19]. To ensure stable results and to avoid over- or underfitting, we implemented a fivefold cross-validation. To identify the most influential features, 12 feature reduction methods were used and compared to reduce the radiomics features to a count of ten. This parameter was chosen to optimally minimize dimensionality as well as the possibility of correlating features reducing the computational performance of the calculation. The utilized methods were the following: ReliefF (RF), Analysis of Variance (ANOVA), Chi-squared (Chi2), Linear Regression (LIR), Logistic Regression (LOR), CART Regression (CARTR), CART Classification (CARTC), Random Forest Regression (RFR), Random Forest Classification (RFC), FisherScore (Fisher), Laplacian and GradientBoost (RB). For each feature selection method, predictions were calculated with 12 classification methods. The chosen classification methods were the following: Neural Networks (NN), Decision Tree (DTREE), Support Vector Machines (SVM), K-Nearest Neighbor (KNN), Random Forest (RF), Logistic Regression (LRC), Gaussian Naive Bayes (GNB), eXtreme Gradient Boosting (XGB), Stochastic Gradient Descent (SGD), Bernoulli Naive Bayesian (BNB), Ensemble Bagged Trees (EBT), and AdaBoost (ABC). All applied methods and their performance in regard to predictions with radiomics features are examined in more detail in our previous work [6]. After the calculations within the folds, overall estimators were calculated with the predictions who lead to the highest weighted F1-Score within the fold. Metrics like the weighted F1-Score, balanced accuracy, and Matthew Correlation Coefficient (MCC) were chosen to optimally compare the outcomes. The feature selection, classification process, and metric calculation were done for each of the folds separately. This radiomics workflow was repeated three times in total to ensure the reproducibility of our calculations. The resulting values were averaged to achieve a general result to compare the calculations for the three cohorts of $\textrm{DB}_{1}$ and $\textrm{DB}_{2}$.

**Fig. 3:**
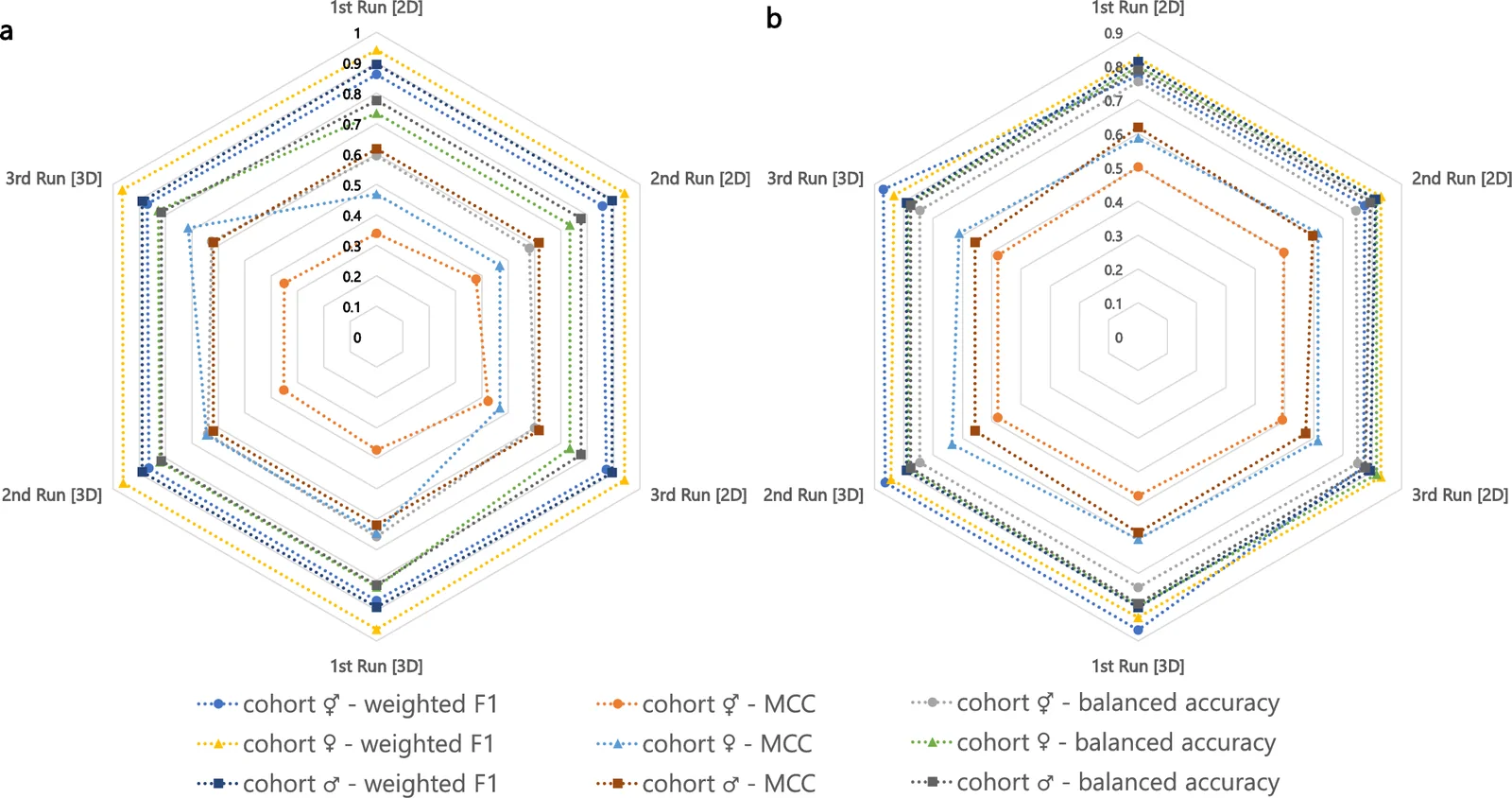
Overview of the highest mean metric values of all three repetition runs for **(a)** $run_{mort}$ and **(b)** $run_{trop}$

**Fig. 4:**
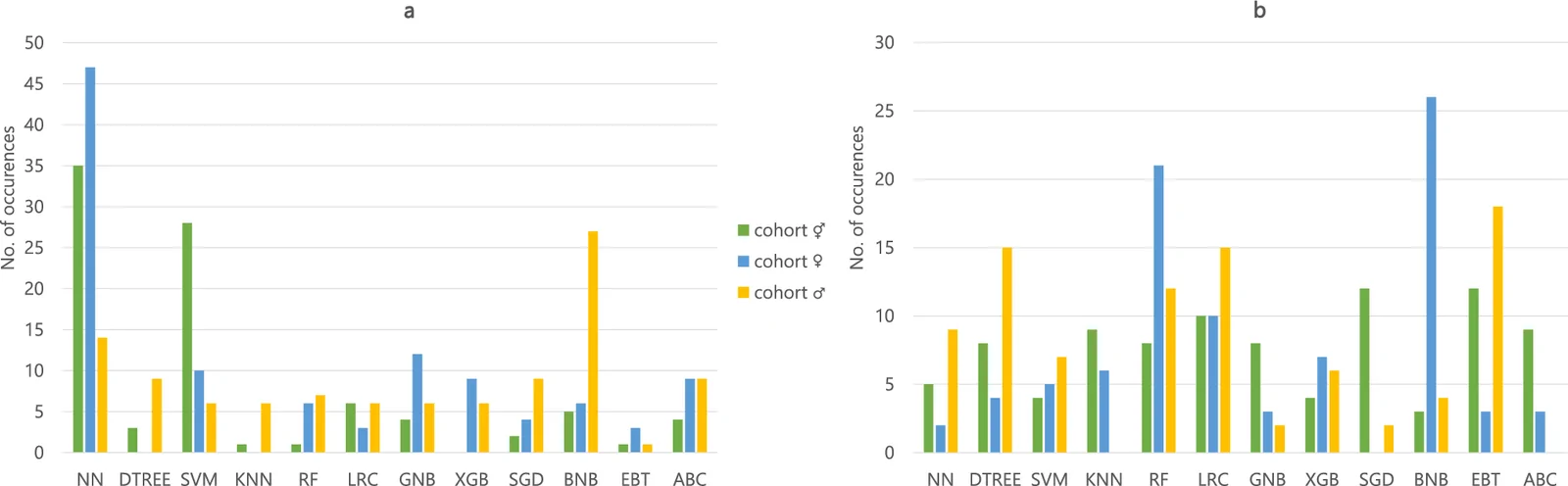
Best-performing classification methods for **(a)** $run_{mort}$ and **(b)** $run_{trop}$ over three repetition runs

## Results

For , the $run3D_{mort}$ lead to the highest results with an weighted F1-Score of 0.9626, an MCC of 0.6685, and a balanced accuracy of 0.8245. $Run3D_{mort}$ also resulted in the highest metric scores for the prediction with , except for the weighted F1-Score. This value was higher in 2D with a score of 0.8941. The highest MCC was 0.6195 and the highest balanced accuracy was 0.8168 for the . The greatest values for  resulted in a weighted F1-Score of 0.8671 for $run3D_{mort}$, an MCC of 0.4974, and a balanced accuracy of 0.7495 for $run2D_{trop}$. In comparison, the highest results for $run_{trop}$ are from  with a weighted F1-Score of 0.8676 and an MCC of 0.6156 for $run3D_{trop}$, as well as a balanced accuracy of 0.8093 for $run2D_{trop}$. An overview about the metric results is shown in Table 1 and in Fig. 2.

We carried out the classification for two additional times to ensure a stable prediction and reproducibility of our study. The **standard deviations** were calculated for all three repetition runs. The smallest standard deviation for each cohort was < 0.0001. The highest standard deviation for  was 0.0188 for the MCC in $run2D_{trop}$, for  0.0327 for the MCC in $run3D_{mort}$ and for the  0.0345 for the MCC in $run2D_{mort}$. In $run2D_{mort}$, the standard deviations for  and  analysis are the same and smaller than the one for the not sex-stratified . In $run3D_{mort}$, the standard deviations for  are smaller than for  and . In comparison, for $run_{trop}$ the standard deviations for  are smaller than for  and . These discrepancies between the metric values and cohorts in the repetition runs are shown in Fig. 3, where the performances with radar charts were compared.

**Fig. 5:**
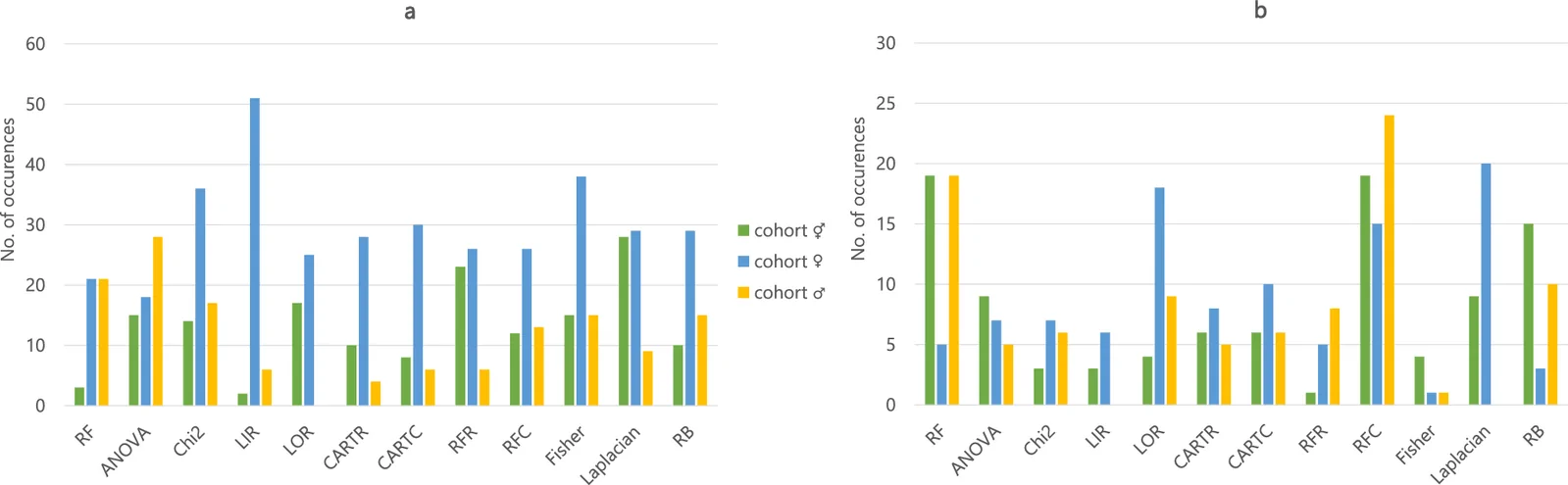
Best-performing feature selection methods for **(a)** $run_{mort}$ and **(b)** $run_{trop}$ over three repetition runs

**Fig. 6:**
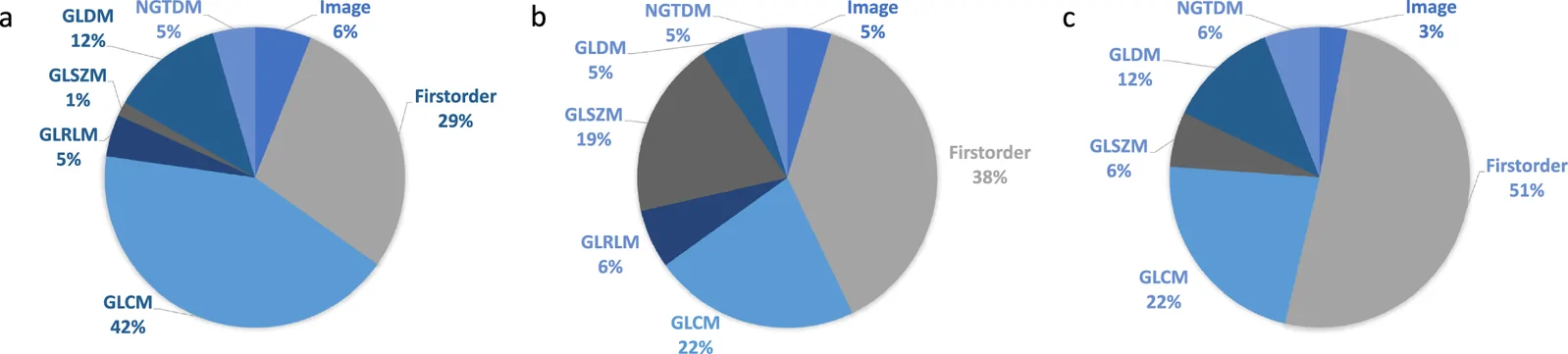
Class percentages of the reduced features for **(a)** , **(b)**  and **(c)**  for 2D

***Classification methods with the highest predictive power*** Figure 4 represents the best-performing classification methods for $run_{mort}$ and $run_{trop}$ for the three cohorts over three repetition runs. The top-performing methods were those that most frequently achieved the highest metrics. These methods are also shown in detail in Table 3.

The methods which achieved the highest results in general are NN, SVM, and LRC for , NN, BNB, and RF for  and BNB, DTREE, and NN for .

***Feature Selection Methods in contrast*** For the comparison of the best-forming feature selection methods, we are visualizing in Fig. 5 the differences between the cohorts for the two target variables ($run_{mort}$ and $run_{trop}$) over three repetition runs. The top-performing methods were those that most frequently achieved the highest metrics as well. These results are additionally perceived in Table 4.

The feature reduction methods which lead to the highest metric results in general are Laplacian, RFC, and RB for , LIR, Laplacian, Chi2, and LOR for  and RF, RFC, and ANOVA for .

**Fig. 7:**
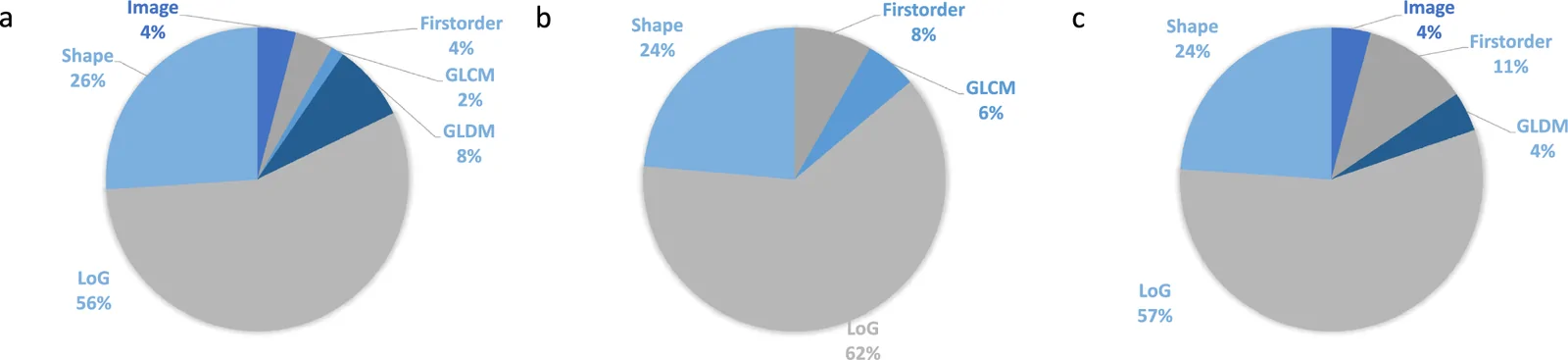
Class percentages of the reduced features for **(a)** , **(b)**  and **(c)**  for 3D

***Analysis of the most important Radiomics Features*** The classes of the most important features were for  in 2D GLCM, Firstorder, and GLDM and in 3D LoG, Shape, and GLDM. For  Firstorder, GLCM, and GLSZM in 2D and Log, Shape, and GLCM in 3D stood out. For  in 2D Firstorder, GLCM, and GLDM and in 3D Log, Shape, and Firstorder were chosen the most. Figures 6 and 7 represent an overview about these results for the three repetition runs combined. The specific features are represented in detail in Appendix A in Table 8.

***Extended dataset results*** To evaluate the generalizability of our methods and calculations, we extended our analysis to the enlarged $\textrm{DB}_{2}$. The metric results are as follows: For , the $run3D_{mort}$ lead to the highest results with a weighted F1-Score of 0.9402, an MCC of 0.4997, and a balanced accuracy of 0.7363. $Run3D_{mort}$ also resulted in the highest metrics for the prediction with . The weighted F1-Score was 0.8961, the MCC 0.5136, and the balanced accuracy 0.7117. The highest values for  were represented with a weighted F1-Score of 0.8832, an MCC of 0.3422, and a balanced accuracy of 0.6482 for $run2D_{mort}$. The highest metric values overall are for the weighted F1-Score and balanced accuracy from $run3D_{mort}$ of  with 0.9402 and 0.7392, respectively, and for the MCC from $run3D_{mort}$ of  with 0.5136. In comparison, the highest results for $run_{trop}$ are from  with a weighted F1-Score of 0.7784 and a balanced accuracy of 0.7392 for $run3D_{trop}$. The highest value for the MCC is 0.4767 from  for $run3D_{trop}$. These results are also shown in more detail in Table 2.

**Table 2:** Highest mean metric values for both target variables, both dimensions, and for the three cohorts of $\textrm{DB}_{2}$

	cohort	cohort	cohort	cohort	cohort	cohort
	${run2D}_{mort}$			${run3D}_{mort}$		
weighted F1	0.9351	0.8671	0.8832	**0.9402**	0.8961	0.8746
MCC	0.4469	0.3845	0.3422	0.4999	0.5136	0.2531
balanced accuracy	0.6608	0.6631	0.6482	**0.7363**	0.7117	0.5629

	${run2D}_{mort}$			${run3D}_{mort}$		
weighted F1	0.7744	0.7222	0.7233	**0.7784**	0.7606	0.7085
MCC	0.4659	0.368	0.3652	0.4742	0.4767	0.3336
balanced accuracy	0.7299	0.6718	0.6701	**0.7392**	0.7351	0.6693

Bold highlights the highest value

Similar to our 200 dataset experiment, we also carried out two additional runs to secure a stable prediction and reproducibility. The smallest standard deviation for each separate cohort was <0.0001. The highest standard deviation was for  0.0224, for  0.0163, and for the analysis of  0.0015. Except for $run2D_{trop}$, the standard deviation for  was lower in all cases. For the comparison of the sex-stratified results,  has lower standard deviations for $run_{mort}$ and higher ones for $run_{trop}$.

## Discussion and limitations

In our previous study [6], we analyzed the overall predictive potential of radiomics features. Consistent with prior findings, $run_{mort}$ resulted mainly in higher values than $run_{trop}$. The metric values and the highest achieving machine learning methods were on the other hand different. Our previous study presented higher metric results. Compared to our previous paper, we utilized in this present study a larger and more diverse dataset and looked into sex-stratified cohorts. We also didn’t implement a data balancing and analyzed on these grounds other metrics. In this study, we utilized databases with a larger number of patient data with a more pronounced imbalanced. Therefore, this decision was made to reduce possible influences of eventually excluding important and including copied patient cases. These influences were considered generally negligible for our first exploratory study, but need to be addressed in the future. Regardless of these differences, the present calculations have proved to be stable with small standard deviations indicating that potential data leakage and overfitting were negligible. Therefore, this study is based on the insights of our previous paper and is considered as an expansion of our established findings.

The metric values of the three cohorts in comparison, which are presented in Table 1 and Fig. 2, indicate a correlation between the best prediction results and the specific sexes. The calculations of  resulted in metric values with overall higher scores compared to the other two cohorts (0.77–31.04%). The largest variance was apparent for the MCC for $run3D_{mort}$. The balanced accuracy and MCC for $run2D_{mort}$ presented an exception with higher metric values for  (4.2–14.86%). On the contrary,  predictions resulted mainly in the lowest metric values. The weighted F1-Score for $run2D_{trop}$ was an exception. These differences can be seen clearly in the radar charts in Figure 3. Lower metric values resulted in smaller and higher values in larger shapes. A consistent form additionally represents a stable prediction over multiple runs and dimensions (2D and 3D). In this regard, for the standard deviations lower values were observed for  and . These results demonstrate an influence of the patients’ sex on the prediction efficiency.  is more proficient in efficiency and stability for the prediction of 30d-mortality and troponin level.  featured lower prediction efficiency in comparison with the calculations with separate sexes.

We observe similarities and differences among the top-performing feature selection and classification methods. One of the methods resulted in the highest metric values for all three cohorts. Other approaches performed comparably for one to two cohorts. These differences are attributable to the diversity in the image properties of the cohorts, which have an influence on the machine learning methods. These distinctions between the image features of the cohorts are apparent in the evaluation of the most important features and feature classes. This is portrayed in Figs. 6 and 7 and in Table 8. One image feature contributed to the predictions of two or three cohorts equally. Other image features were different depending on the dimensionality and patients’ sex. Regarding the feature classes, we detected differences after the two most frequently occurring groups and in respect to the occurrence rates. The class distributions indicate different relevance of the thrombus properties for the cohorts. For , the information about the texture of the thrombus defined by the GLSZM and LoG features, for example how coarse the thrombus area is, is relevant. For , the voxel intensity defined by the Firstorder features exhibits higher importance. Therefore, the composition of the thrombus is a defining factor. In contrast, the shape and heterogeneity of the thrombus, defined by the Shape and GLCM features, have more influence on the predictions of . Based on these results, we conclude that the most contributing features are different depending on the sex of the patient. This can be attributed to many factors, which could be of biological nature. A comprehensive analysis of these differences is beyond the scope of this study. For a better insight, these results need to be studied and additionally evaluated by a physician expert.

We observe the same trends while comparing the metric values of $\textrm{DB}_{1}$ and $\textrm{DB}_{2}$. For $\textrm{DB}_{2}$, the  resulted in the highest metric values in general with the exception of the balanced accuracy during $run2D_{mort}$ and the MCC during the 3D run. For these two metrics  presented higher values.  featured the lowest results. Solely one exception was observed with the weighted F1-Score for the 2D calculations. The lower prediction efficiency of  is therefore consistent with the results of $\textrm{DB}_{1}$. The best-performing methods for the feature selection and classification stayed consistent with $\textrm{DB}_{2}$, with only a few additional methods showing comparable promise. These observations demonstrate that, independent from the number of patient data, the identified methods are considered to have a high prediction potential in general. The classes of the most presenting features in $\textrm{DB}_{2}$ were the same between the cohorts, but contributed to the prediction to varying degrees. Therefore, it can’t be clearly stated which properties of the thrombus have higher influence for which cohort. This distinction to the results of our initial database is assumed to arise due to the variations in the highest achieving feature selection methods as well as the different number of female and male patients compared to $\textrm{DB}_{1}$. These outcomes demonstrate that our methods can be extended to datasets, which represent a larger database and are not manually segmented and checked. Our methods and overall findings are therefore considered generalizable and applicable to other patients’ data.

To the best of our knowledge, there are no similar studies to this sex-stratified analysis. A direct comparison of our findings with previous research results is hardly feasible. However, other studies analyze the sex differences of APE patients in regard to their deviations in treatment and outcome. The prospect of the patients’ sex representing an independent indicator for APE predictions is studied as well. No certain factor or finding regarding the specific influence of the patients’ sex on APE directly is mentioned. Other papers indicate a prediction potential of female patients’ data for the mortality or death at admission [10, 13], while others disprove these hypotheses and determine no significant correlation between the patients’ sex and their mortality [11, 12]. In relation to the blood level of troponin, Jankowiak et al. [20] suggest an observable correspondence to the patients’ sex. In general, previous studies identified no clear distinct indicator about the variety of the influence of the patients’ sex on APE predictions. The differences of the sexes in regard to risk factors, systematic symptoms, clinical representation, diagnosis, and treatment as well as outcomes of APE are proved to be certain [11, 21] and suggest differences in the patients’ image information, which we demonstrate in our findings.

This study is restrained due to the limited size of the dataset. On account of an automatic segmentation, we utilized around 400 patient cases. For machine learning processes, a further extended database with increased diverse patient information will tend to result in more distinct findings. A larger number of cases additionally counteract the data imbalance regarding the target variables to ensure more reproducible outcomes throughout the training of the classifications. Similar to our previous study [6], we include thrombi of different areas in the lung arteries to minimize the effect of possible artifacts induced by contrast agents in the Vena Cava. This ensures in all cases that parts of the thrombi aren’t affected by an artifact. Our results are therefore to the best of our knowledge reproducible and reliable. The metric values for $\textrm{DB}_{2}$ are lower than the results of the initial. This outcome has various causes. The automatic segmentation presents a Dice similarity coefficient up to 90% compared to a ground truth and was not manually checked for all patient cases. $\textrm{DB}_{2}$ additionally consists of a different sex distribution with a larger number of male patients. However, the general findings regarding the influence of the patients’ sex on our predictions are comparable to $\textrm{DB}_{1}$ and still prove that the trends we identified are applicable on a differently prepared and larger dataset from the same study cohort. Since $\textrm{DB}_{1}$ and $\textrm{DB}_{2}$ apply the analysis on the same source population, a validation with an independent multicenter cohort will be needed in the future to further verify our results.

## Conclusion

In our study, we analyzed the predictive power of the patients’ sex in regard to mortality and troponin levels of APE patients while utilizing radiomics features of the pulmonary artery thrombus. Comparing the results of the cohorts, differences in their prediction efficiency were apparent.  presented the highest and most stable calculation with the largest metric value variance of 31.04% followed by .  achieved lower results and appeared less robust. The best-performing feature selection and classification methods revealed to be different throughout the cohorts with the exception of CARTC and NN. The specific radiomics features, which contributed to the calculations the most, presented different results for the cohorts as well. For , the coarseness of the thrombus proved to be of importance, whereas for  the composition and for  the shape and heterogeneity of the thrombus further impacted the results. We demonstrated an influence of the patients’ sex combined with the arterial thrombus image information on the prediction of mortality and troponin level for APE patients. This outcome was additionally replicated with anextended dataset from the same source population, which was automatically segmented and displayed a different sex distribution. These result could therefore help future studies to identify further differences between the representation and image information of APE patients in regard to their sex and support APE diagnosis and treatment.

**Table 3:** Best-performing classification methods for the metrics for the three cohorts ($run_{mort}$ and $run_{trop}$)

	$run_{mort}$	$run_{trop}$
Combined		
	SVM · KNN · LRC · SGD	ABC · GNB · NN · SVM · XGB
Female		
	NN · GNB · XGB · SGD	RF · KNN · NN · ABC
Male		
	BNB · NN · DTREE	EBT · RF · NN · LRC

**Table 4:** Best-performing feature reduction methods for the metrics for the three cohorts ($run_{mort}$ and $run_{trop}$)

	$run_{mort}$	$run_{trop}$
Combined		
	Laplacian · RFR · ANOVA	RF · RB · ANOVA · Laplacian
	· CARTR · CARTC · RB	· Fisher · LIR · CARTR+C
Female		
	LIR	LOR RFC · Laplacian · CARTR
	· Fisher	· CARTC · ANOVA
Male		
	ANOVA · RF · RB · Chi2	RFC · RB · RF · ANOVA · CARTC

**Table 5:** Patient information of the source population with mean and standard deviation (std) values

Clinical parameters	Labels	Value	Male	Female
Sex			310	221
Mortality	0	466	263	203
	1	65	47	18
Troponin Level	0	131	88	96
	1	213	117	43
sPESI	Mean	1	1	1
	std	1.05	1.05	1.05
Age	Mean	65	64	67
	std	16	14	17
	Min / Max	15 / 96	15 / 96	18 / 96
Size [cm]	Mean	173	179	165
	std	10	8	7
Weight [kg]	Mean	87	91	82
	std	22	21	21
Administration of catecholamines	Yes	57	39	18
	No	473	270	203
RR syst.	Mean	134	133	134
	std	28.54	29.54	27.13
Hemodynamical instability	0	477	273	204
	1	35	23	12
RR diast.	Mean	80	80	80
	std	17.81	17.51	18.2
Heart rate	Mean	96	97	96
	std	24.89	25.26	24.38
Lactate	Mean	2.49	2.52	2.45
	std	2.47	2.31	2.68
IVC-Reflux	Mean	1	1	1
	std	0.9	0.91	0.89
RV diameter [mm]	Mean	40	42	38
	std	9.2	9.2	8.57
LV Diameter [mm]	Mean	38	40	36
	std	9.62	9.79	8.78
RV/LV ratio	Mean	1.14	1.13	1.14
	std	0.45	0.45	0.46
Left-sided nasal septum deviation [mm]	Mean	1	1	1
	std	2.93	3.11	2.66

## Figures and Tables

**Fig. 8:**
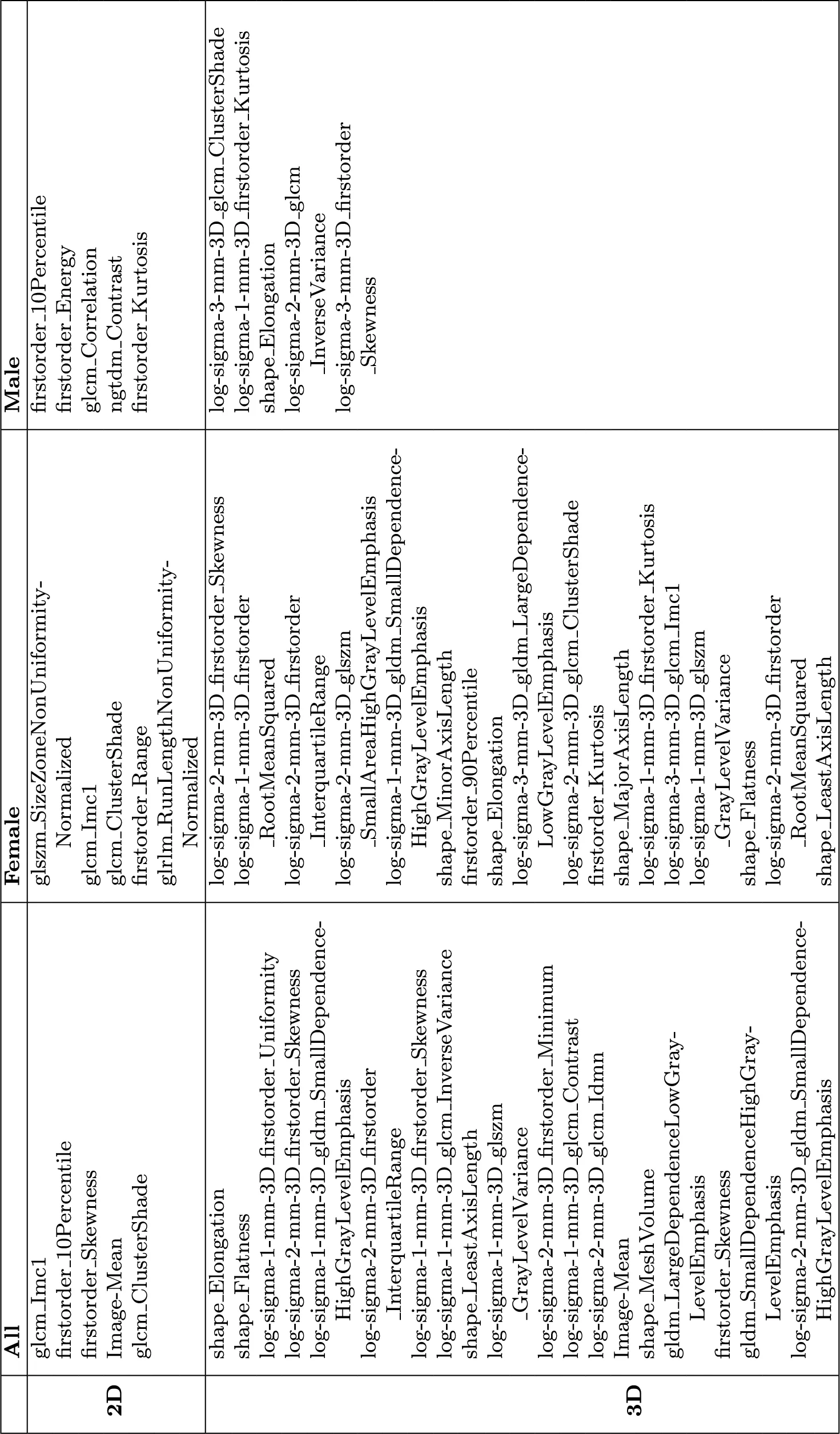
Most often reduced features for all three cohorts in comparison separated by the dimensionality

## A Appendix

See Tables 3, 4, and 5 Figure 8
